# sEEG-based brain-computer interfacing in a large adult and pediatric cohort

**DOI:** 10.1088/1741-2552/ae2955

**Published:** 2025-12-30

**Authors:** Michael A Jensen, Gerwin Schalk, Nuri Ince, Dora Hermes, Greg A Worrell, Peter Brunner, Nathan P Staff, Kai J Miller

**Affiliations:** 1 Department of Neurosurgery, Mayo Clinic, Rochester, MN, United States of America; 2 Neurotechnology Innovation and Impact Institute, West China Xiamen Hospital of Sichuan University, Xiamen, People’s Republic of China; 3 Department of Electronic & Electrical Engineering, University of Bath, Bath, United Kingdom; 4 Department of Biomedical Engineering, Mayo Clinic, Rochester, MN, United States of America; 5 Department of Neurology, Mayo Clinic, Rochester, MN, United States of America; 6 Department of Neurosurgery, Washington University School of Medicine, St Louis, MO, United States of America; 7 Department of Pediatrics, Mayo Clinic, Rochester, MN, United States of America

**Keywords:** brain computer interfacing, stereoelectroencephalography, motor BCI

## Abstract

**Objective.:**

Stereoelectroencephalography (sEEG) is a mesoscale intracranial monitoring technique that records from the brain volumetrically with depth electrodes. sEEG is typically used for monitoring of epileptic foci, but can also serve as a useful tool to study distributed brain dynamics. Herein, we detail the implementation of sEEG-based brain-computer interfacing (BCI) across a diverse and large patient cohort.

**Approach.:**

Across 27 subjects (15 female, 31 total feedback experiments), we identified channels with increases in broadband during hand, tongue, or foot movements using a simple block-design screening task. Subsequently, broadband power in these channels was coupled to continuous movement of a cursor on a screen during both overt movement and kinesthetic imagery.

**Main results.:**

26 subjects (29 out of 31 feedback conditions) established successful control, defined as more than 80 percent accuracy, during the overt movement BCI task, while only 12 (of the same 31 conditions) achieved control during the motor imagery BCI task. In successful imagery BCI, broadband power in the reinforced control channel(s) in the two target conditions separated into distinct subpopulations. Outside of the control channel(s), we demonstrate that imagery BCI engages unique subnetworks of the motor system compared to cued movement or kinesthetic imagery alone.

**Significance.:**

Pericentral sEEG-based motor BCI utilizing overt movement and kinesthetic imagery is robust across a diverse patient cohort with inconsistent accuracy during imagined movement. Cued movement, kinesthetic imagery, and feedback engage the motor network uniquely, providing the opportunity to understand the network dynamics underlying BCI control and improve future BCIs.

## 1. Introduction

A brain-computer interface (BCI) is a technology that enables direct communication between the brain and an external device by translating neural activity into digital output signals. BCI control has historically been facilitated by technologies that capture neural activity generated within the brain’s convexity. These include scalp electroencephalography (EEG) [1, 2], magnetoencephalography [3], electrocorticography (ECoG) [4, 5], micro-ECoG [6, 7], and single neuron recordings [8, 9]. In this study, expanding on an anecdotal study in two subjects [10], we aim to explore whether neural populations can be volitionally modulated—quantified by high frequency broadband power -throughout the brain’s depths to control a BCI using stereo EEG (sEEG) depth electrodes. If true, these recordings could be used in conjunction with, and inform the placement of, high-density arrays within these populations (e.g. with the Neuropixel), suggesting the possibility of more robust control through tandem measurements across the brain’s volume.

sEEG is a mesoscale measure of brain activity that uses intracranial depth electrodes [11–13]. Like ECoG, it measures the summation of local field potentials generated by thousands of neurons surrounding the recording electrode, and correlates to single-unit spiking [14, 15]. Unlike ECoG, sEEG is not limited to the surface of the cortex, and allows for sampling from distant cortical and subcortical regions [16, 17]. While the current clinical purpose of sEEG is to record the onset and propagation of seizures in patients with drug-resistant epilepsy, it can be an excellent opportunity to study distributed brain circuitry when patients elect to participate in experiments during their stay in the hospital.

Recent studies demonstrated that the neural markers of overt or imagined movement are present outside primary motor cortex. These include the thalamus [18], subthalamic nucleus [19], basal ganglia [20], supramarginal gyrus [21, 22], insula [21, 23, 24], posterior parietal cortex [25, 26], pre-motor cortex [22], and even the temporal lobe and hippocampus [23]. Although the direct evidence for this is limited, these non-primary cortices contain information regarding movement planning, coordination, and execution that complements the neural content within primary motor cortex [26, 27].

In this manuscript, we demonstrate that sEEG-based BCI can be implemented across a diverse set of brain regions and patient populations, positioning sEEG-based BCI as a powerful approach for investigating distributed cortical and subcortical network dynamics. By enabling sampling throughout the brain’s volume, sEEG-based BCIs naturally complement many modern approaches [9, 28–30] that rely on high-density recordings from select cortical regions, thereby creating a richer foundation for future BCI design and application.

## 2. Materials and methods

### 2.1. Ethical statement

The study was approved by the Institutional Review Board of the Mayo Clinic (IRB 15–006 530) and conducted according to the guidelines of the Declaration of Helsinki. Each patient or their parental guardian provided informed consent as approved by the IRB.

### 2.2. Subjects

Twenty-seven patients (12 females, 6–46 years of age—table 1) participated in this study after implantation with sEEG electrode leads for the treatment of drug-resistant epilepsy. Subjects were included in the study if at least one channel was identified as having a significant increase in high frequency band power (broadband) during movement periods compared to rest in our motor screening task [31]. 10–18 leads (10, 12, 15, or 18 electrode contacts per lead) were implanted per subject, with a total of 5664 contacts across all 27 subjects. Electrode planning was performed by the clinical epilepsy team based on brain imaging, typical semiology, and scalp EEG studies. The planning of electrode locations was guided solely by clinical decision-making and was not modified to accommodate research. All experiments were performed in the adult epilepsy monitoring unit or pediatric intensive care unit at the Mayo Clinic in Rochester, MN. All T1 MRI sequences were de-identified prior to publication [32, 33] to assure anonymity of subjects.

### 2.3. Hardware, lead placement, electrode localization, Re-referencing, channel visualization

Platinum depth electrode leads (DIXI Medical) were 0.8 mm in diameter with 10–18, 2 mm length circumferential contacts separated by 1.5 mm. Voltage timeseries were recorded using a g.HighAmp amplifier at 1200 Hz during the motor task and 2400 Hz during the BCI task. An electrode located in white matter was selected as the hardware ground. Surgical targeting and implantation were performed in the standard clinical fashion [13]. Anatomic locations of electrodes were determined by co-registration of post-implant CT scan to pre-implant MRI. Pre-operative T1 MRIs were aligned to the anterior and posterior commissure stereotactic space using VistaSoft [34], and co-registered to the post-implant CT using SPM12 [35]. Electrodes were plotted in the merged MR-CT space using a custom open source MATLAB toolbox (‘SEEGVIEW’) [36].

All data were re-referenced to a bipolar montage to represent differences in local field potentials at *two* adjacent electrode contact sites, referred to as channels. Two electrode contacts generate a single channel if and only if they are 1.5 mm from one another, on the same lead, and within the same lead segment for segmented leads. All figures represent these channels, which is calculated as the interpolated position between the center of each electrode contact. Plots include volumetric brain renderings and axial, saggital, & coronal slices generated using SEEGVIEW [36]. Of note, channels are projected to the nearest slice, and therefore do not perfectly represent a channel’s position.

### 2.4. Motor and kinesthetic imagery screening tasks

We performed a motor screening in all subjects for the purposes of mapping functional representation of movement across the sEEG montage [31]. This task involved: 1) opening and closing of the hand; 2) side-to-side movement of the tongue with mouth closed; and 3) alternating dorsi- and plantar flexion of the foot (contralateral to the hemisphere(s) with peri-central sEEG electrode coverage). Subjects were cued to perform these simple self-paced movements using images of a hand, tongue, or foot at a pace of one repetition per second, and remain still for 3 s during interleaved rest periods (blank black screen). Twenty trials (3 s in duration) of each movement type were shuffled in random order. This task was chosen based upon prior work that produced clear results in brain surface recordings [31, 37–39]. In a subset of patients, we repeated the same task except that we asked the patients to use kinesthetic imagery instead of overt movement. In brief, kinesthetic imagery involves concentrating on the internal sensations of motion—such as muscle tension and joint position—rather than on how the movement appears externally. Subjects who did not participate in the imagery task did so because either 1) they requested to not participate (*n* = 2), 2) there was insufficient time left by the clinical schedule to perform this task (*n* = 3), or 3) behavioral observation indicated that the subject did not properly engage in a task (*n* = 6). We used BCI2000 software for data acquisition, stimulus presentation, and data synchronization [40, 41], with stimuli presented on a 53 × 33 cm screen, 80–100 cm from the face, with the cues spanning approximately 30–40° of the visual field.

### 2.5. Post-hoc signal processing and analysis

We performed all analyses in MATLAB. To assure accurate segmentation of data into hand, foot, and tongue movements and rest periods, we recorded surface EMG from the forearm, shin, and chin respectively during all experiments synchronized to intracranial time series. These EMG time series were band-passed from 25 to 400 Hz and rectified to allow visual demarcation of movement onset and offset [31, 38]. Specifically, the onset and offset of movements were manually demarcated based on the sharp increases and decreases in EMG signal such that sEEG time series could be epoched into movement and non-movement periods based on these markings. Within each movement trial, we calculated average power spectral densities (PSDs) from 1 to 300 Hz every 1 Hz using Welch’s averaged periodogram method with 1 s Hann windows to attenuate edge effects and 0.5 s overlap [42]. The averaged PSD for each movement or rest trial was normalized to the global mean across all trials. The PSDs were normalized in this way since brain signals of this type generally follow a 1/f power law and shape [15], so that lower frequency features dominate in the absence of normalization. From each of these normalized single-trial PSDs, averaged power in a high-frequency band (65–115 Hz—representing broadband power) was calculated for subsequent analysis as previously described [31, 38]. This band was chosen as it captures broadband activity above most oscillations and avoids ambient line noise at 60 and 120 Hz.

For each bipolar re-referenced channel, we calculated signed cross-correlation values (*r*^2^) of the mean spectra from 65–115 Hz for each movement modality. The *r*^2^ value of each channel was determined by comparing mean power spectra between rest and movement trials separately. To minimize beta rebound cross-effects [43], movement trials of each type were only compared with rest trials that followed that same movement type. The sign of each *r*^2^ indicates whether power is increasing or decreasing with movement, as illustrated by red and blue circles, respectively, in each figure. From here the bipolar channels with the highest *r*^2^ values were chosen as controls channels in subsequent BCI experiments.

### 2.6. BCI task

In our BCI task, cursor control on a screen was coupled to high frequency power changes recorded in preselected bipolar channel(s), and subjects attempted to control the cursor in one dimension toward a target. Using BCI2000 [40], we applied a real-time spectral estimator to incoming signals using an autoregressive model [44] of the input, operating like a Fast Fourier Transform with a limited number of coefficients. A linear classifier was applied to the feature space of 90–130 Hz power in the channel(s) chosen for BCI to allow for cursor control. During the first experimental run (5–10 trials), BCI2000 adapted this classifier based on the mean and variance of the previous 30 s of incoming data (data buffer). This run was not included in the analysis. The threshold, *P*_0_, was then set to the mean of the data buffer, and the velocity, g, was set to the inverse square root of the variance of the data buffer (figure 3). After the initial run, these parameters were kept fixed for the remainder of the experiment to avoid any confound from classifier adaptation during the subject’s learning to exert BCI-control over the cursor.

Feedback was delivered to channels that demonstrated the highest *r*^2^ values [38] during the motor screening task (figure 2). This channel used in imagery and motor BCI were always identical (figures 5(b) and (c)). Although assessment of brain activation was kept uniform for all analyses at 65–115 Hz to avoid line noise and its second harmonic, 90–130 Hz power was chosen for feedback in order to confirm the ability to span 120 Hz line noise *in vivo* after bipolar re-referencing and to avoid contamination by intrinsic gamma oscillations [45]. If multiple channels were used to control the BCI, then the average broadband power across the *n*channels were used to control the cursor. Note that subjects 1,9, and 11 were ⩽10 years old and therefore did not participate in the imagery BCI task.

Prior to the first experimental run, subjects were instructed to perform movement (motor BCI—hand open/close) or kinesthetic imagery (imagery BCI) depending on the position of a rectangular target (top/bottom, left/right). For example, when the target appeared at the top of the screen the subjects might open and close their hand, but remain still when the target appeared on the bottom of the screen. Targets associated with movement or imagined movement are referred to as active targets, while those associated with rest are passive targets. Note that oftentimes controlling a cursor toward the passive targets requires a cognitive suppression of the control cortex, but since they are at rest externally, the term passive is used. At the start of each trial, a rectangle appeared at the top or bottom of the screen for 2 s. Subjects were cued to move or imagine moving once a cursor appeared on the screen (figure 3). In each trial, subjects were allowed 5 s to move the cursor to the target as prolonged trials can lead to task fatigue and broadband power diminishes when cortical activation is repeated without rest [39]. Each run was limited to two minutes. As such, the longer each trial, the fewer trials were performed per run. Cursor position was updated at a rate of 10 Hz throughout the trial. If the target was not hit during those 5 s, this trial was not considered in the accuracy calculation—potentially inflating accuracy measures—and a new trial began. The inter-trial rest interval was 3 s and each experimental run consists of 2 min of consecutive trials, allowing subjects to complete as many trials as possible while accuracy was logged. The first run was for calibration, such that the BCI2000 adaptation algorithm could fully estimate the mean and variance of the power changes in the reinforced channel as subjects alternated between movement and rest.

## 3. Results

### 3.1. Movement

**Figure 1.**
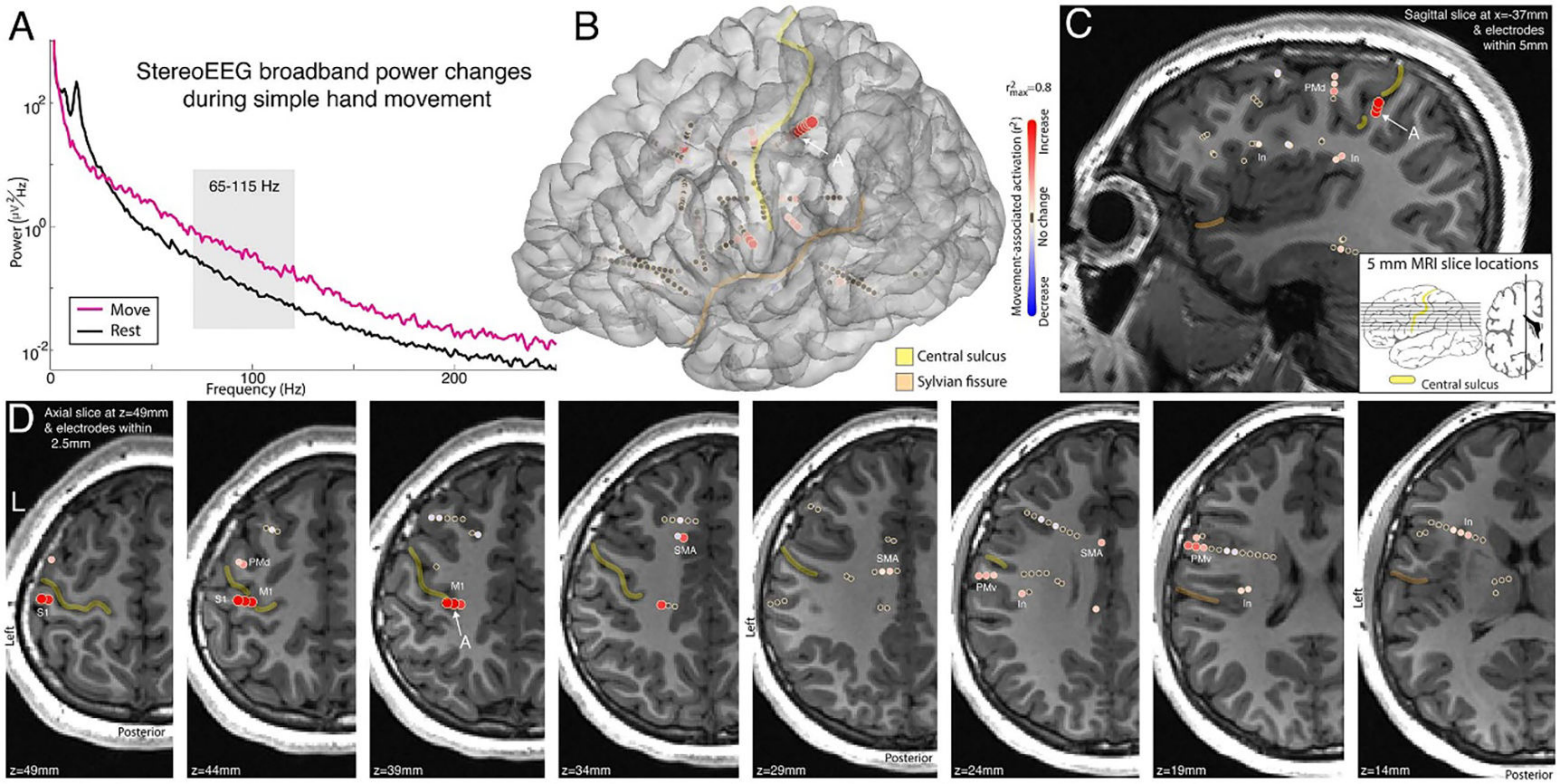
sEEG recordings during movement—Subject 1. (A) Power spectrum from a SEEG channel at the sulcal base of primary motor cortex during hand movement (pink) and rest (black), from the recording site noted in panels (B)–(D). (B) Broadband power (approximated by 65–115 Hz band) increases during movement compared to rest. (C) Sagittal slice showing channels with broadband power (approximated by 65–115 Hz band) increases during movement compared to rest. (D) As in (C), but for axial slices and channels within 2.5 mm. Activation maps for movement are shown in the central colorbar (signed *r*^2^, scaled to 1 maximum, with red/blue reflecting broadband power increase/decrease with movement). Yellow and peach in (B)–(D) indicate the central & Sylvian fissures. Note the simultaneous measurement of M1, PMd (dorsal pre motor), PMv (ventral pre motor), Insula (In), SMA, and S1 (primary sensory), which all show movement-associated broadband power increases.

**Figure 2.**
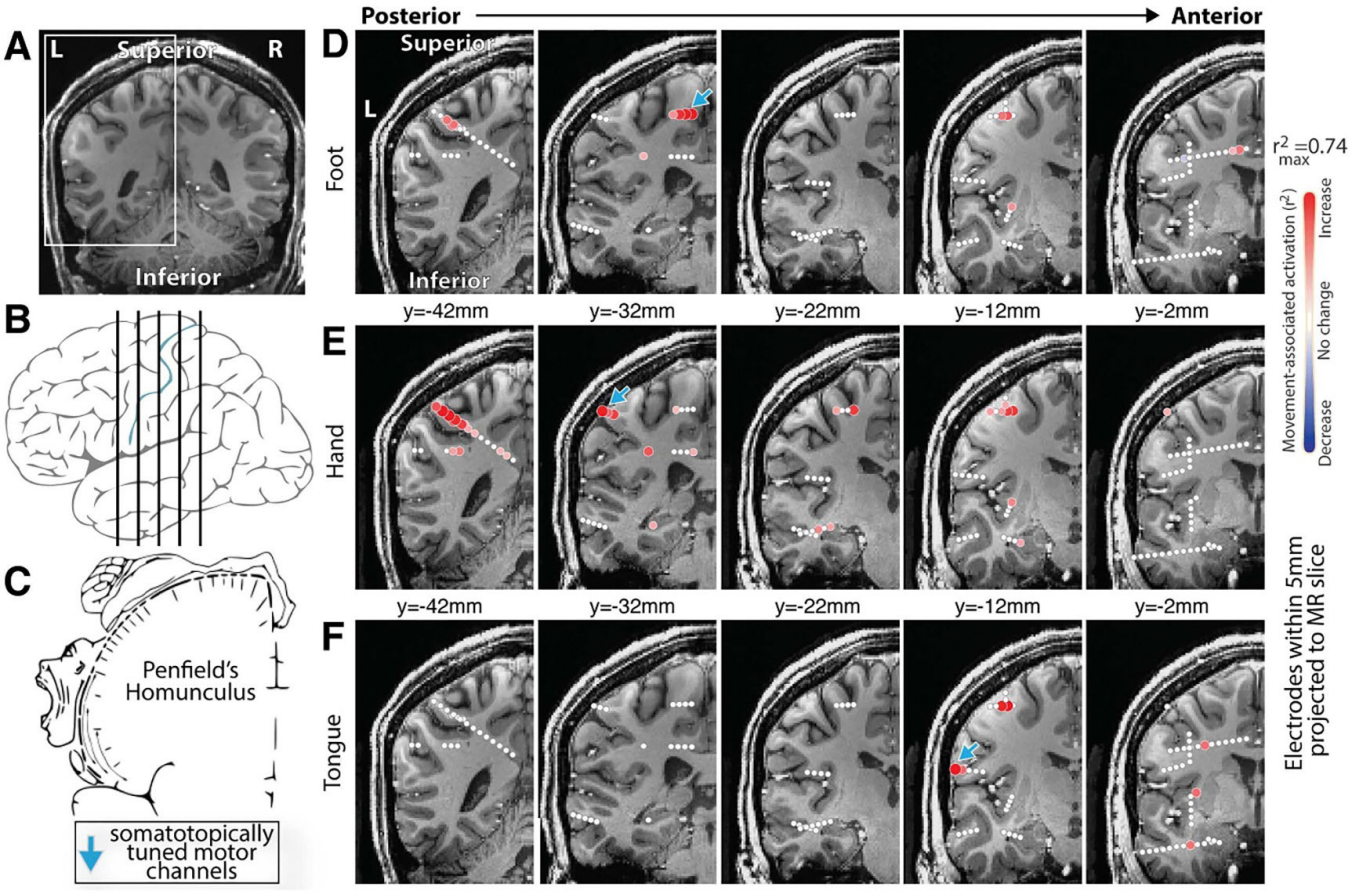
The homunculus in 3 dimensions—Subject 2. SEEG allows us to sample the volumetric structure of the homunculus electrophysiologically. (A&B) Locations of coronal insets in (D)–(F). (C) Reproduction of Penfield’s classic motor homunculus (Wikipedia.org). (D) Comparison of blocks of foot movement vs rest from an SEEG array, plotting movement-associated broadband (65–115 Hz) change. (E)&(F) As in (D), for hand and tongue movement. Note that the classic 2-dimensional homunculus extends into the brain depths, reflecting the volumetric nature of motor representation.

After participating in our motor screening task, changes in the PSD within each sEEG channel were compared between movement and rest periods, and as in previous studies [4, 38]. Movement resulted in suppression of oscillatory activity and an increase in broadband power (figure 1). As broadband power is correlated to local neuronal activity [46], it served to localize functional representation of movement across the sEEG montage (figure 1). Germane to our goal of implementing a BCI, this enabled the identification of the somatotopically tuned cortical regions that could generate the control signal in a closed-loop feedback task (figure 2).

### 3.2. Imagery

A subset of 16 subjects repeated the movement task, but were instructed to kinesthetically imagine performing the cued movement [4, 47–49]. As demonstrated in ECoG [4], kinesthetic imagery produced an increase in broadband power within motor regions just as during movement (figure 6).

### 3.3. BCI closed-loop feedback

**Figure 3.**
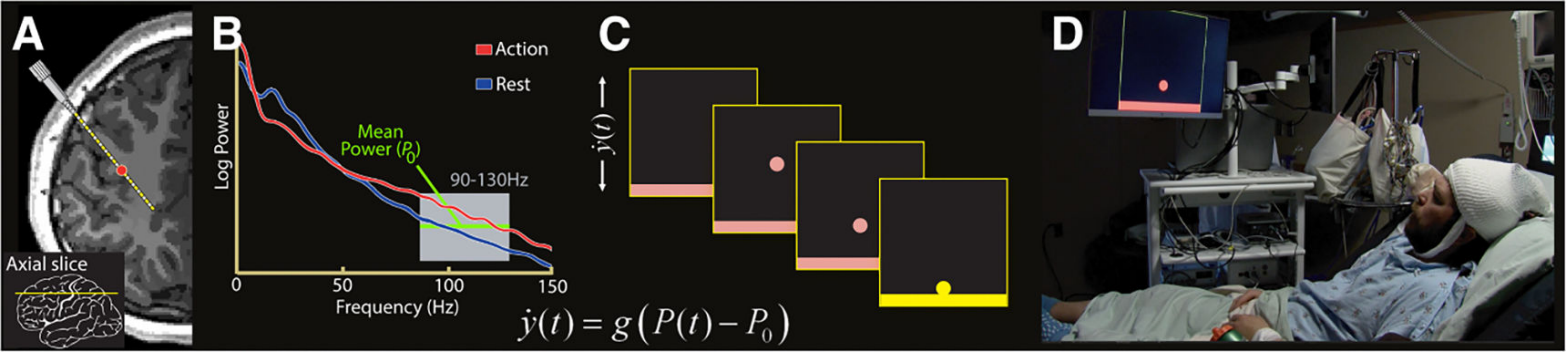
Schematic of online BCI feedback (A) Feedback is provided to a bipolar channel active during movement. This is an example channel in the precentral gyrus. (B) Power from 90–130 Hz in the channel chosen in A determines the direction and velocity of the cursor on the screen. The difference between spectral power from 90–130 Hz and *P*_0_ is coupled to cursor movement, while *g* is the gain coefficient, determining cursor velocity. (C) Targets are displayed prior to cursors to cue movement or rest, and subjects attempt to direct cursors toward the rectangular target. (D) Subjects perform the BCI within their bed, viewing a monitor 80–100 cm from their head, spanning # degree of their visual field.

**Figure 4.**
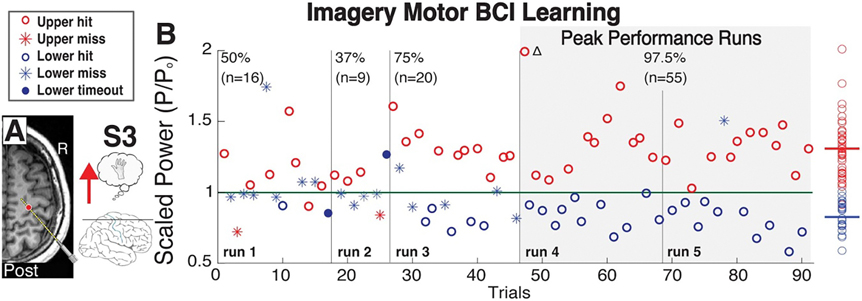
Learning During BCI feedback—Subject 3 (A) Feedback was provided to a bipolar channel in the precentral gyrus of subject 3. (B) High frequency band power is shown across 5 consecutive runs. The subpopulations of power during trials of opposing targets gradually separated across the learning process until an accuracy of 97.5% was obtained (average accuracy across last two runs). Color (red/blue represents the identity of the target shown; asterisks represent misses; hollow circles represent hits; filled circles represent invalid trials lacking a hit or miss.

**Figure 5.**
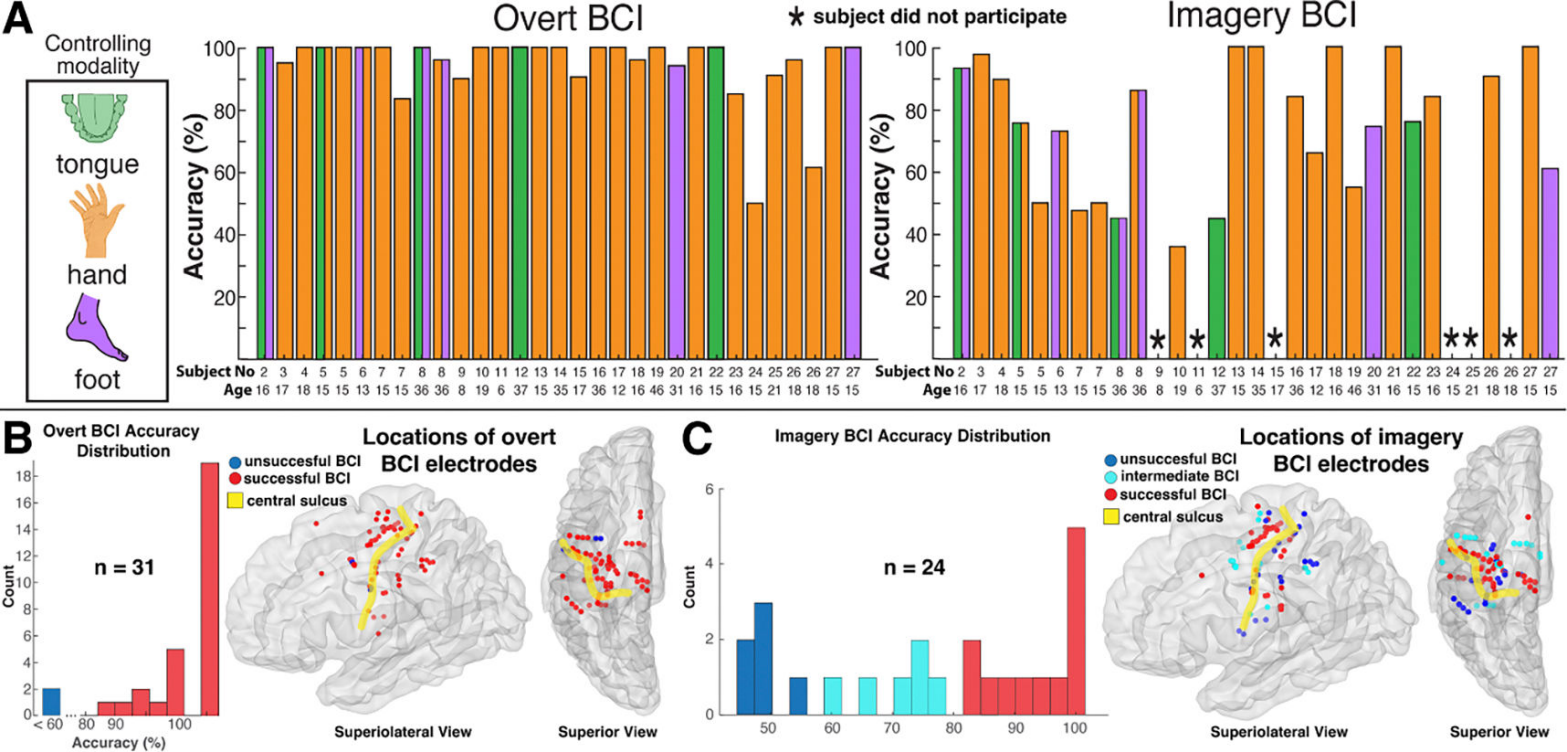
Overt and Imagery BCI accuracy across subjects. (A) Both overt (left) and imagery BCI accuracies are displayed for each subject. In some cases, subjects performed multiple BCIs that differed in either the pre-selected sEEG channels, controlling modalities, or both. Each BCI within these subjects were assigned a unique bar. (B) Distribution of accuracies during overt BCI (left) with locations of BCI controlling electrodes transformed to the left hemisphere of the MNI152 brain. (B) As in (B), but during imagery BCI. Note the control channel for subject 24 was in white matter.

Successful BCI control was defined as runs in which the cursor was moved to the correct target within the alloted 5 s in ⩾80% of trials. Control channels were chosen based on the changes in broadband power during the motor screening tasks, and it was modulation of this power that controlled the speed and 1-dimensional movement of a cursor on a computer monitor a few feet from the patients’ head (figure 3). As represented by subject 3, learning imagery BCI leads to the gradual separation of the average 90–130 Hz power within control channels between trials with opposing targets (figure 4). Twenty-five of twenty-six subjects (96% of total) established successful control of the overt BCI. Of these twenty-five subjects, twenty attempted to perform imagery BCI, with four subjects attempting two separate imagery BCIs for a total of twenty-four. Separate BCIs indicate experiments within the same subject with unique movement modalities, control channels, or both. Movement modality, control channels are referred to as the ‘conditions’ of the BCI. Among these subjects, twelve (60% of total) were able to attain successful BCI control, and six (30% of total) controlled the cursor with an average accuracy of more than 50% (table 1, figure 5). The location of the control channel varied across patients, but the majority (64%) of control channels were within the precentral gyrus (PCG). Although control channels within the PCG may be assumed to lead to the highest accuracies, several channels within the PCG did not allow for control of imagery BCI ⩾60% (figure 5).

### 3.4. Differential cortical engagement across tasks

**Figure 6.**
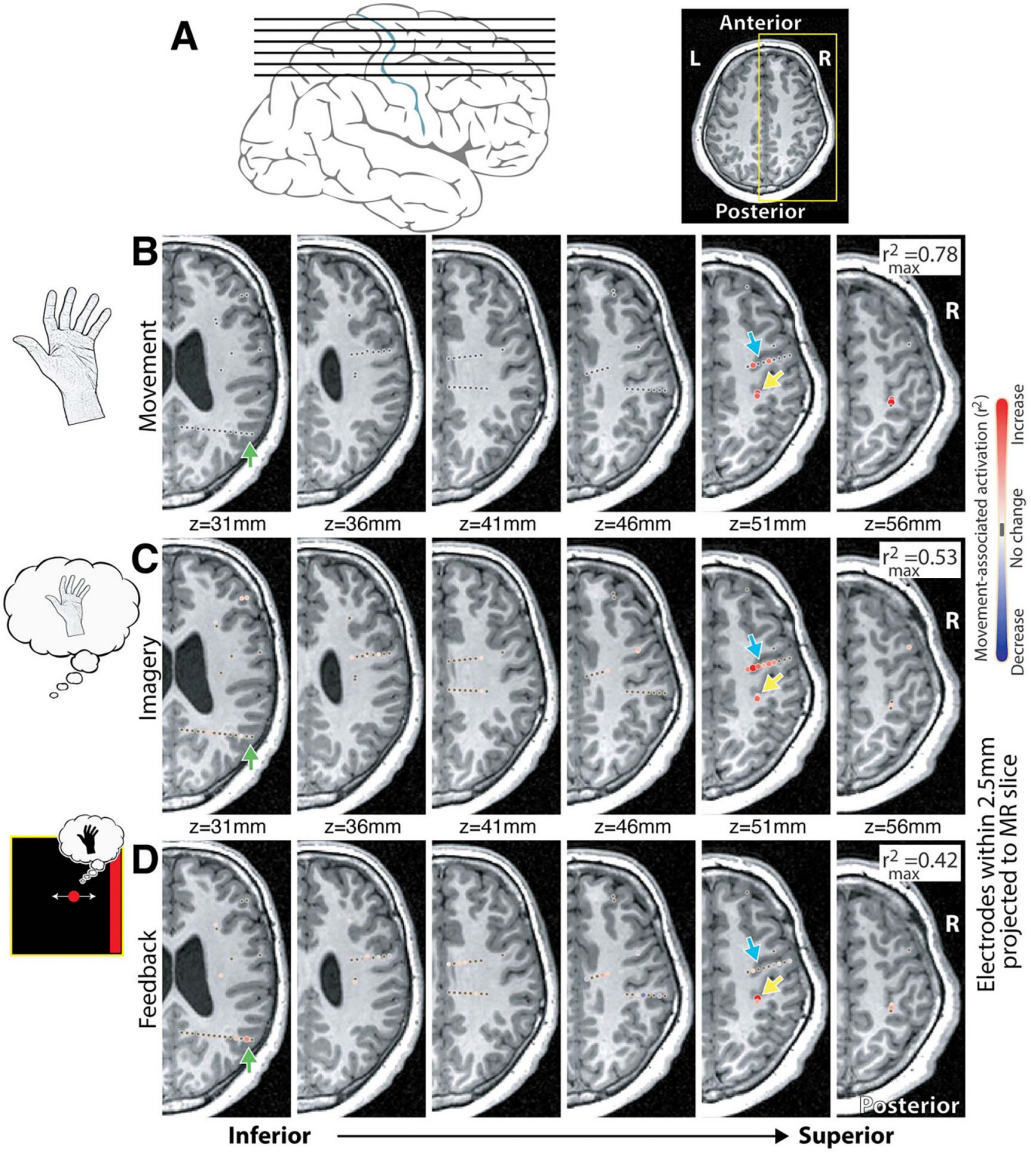
Hand movement, movement imagery, and one-dimensional BCI cursor control using sEEG—Subject 4. (A) Axial insets in (B)–(D) are as shown here. (B) *r*^2^ maps of hand movement vs rest, broadband 65–115 Hz power, as in figure 1(D). (C) Hand movement kinesthetic imagery in the same patient. (D) Map of left hand imagery-based cursor control, comparing left-to-right target presentation times (cursor velocity linked linearly to 65–115 Hz power from M1 site indicated by yellow arrow). Note the selective augmentation in recruitment of the PMd (dorsal pre motor—blue arrow) during movement imagery, and the PRR (parietal reach region -green arrow) activity selectively during BCI, but not during movement or imagery.

Although successful BCI control necessitates broadband power modulation within the reinforced channels, activity patterns within the rest of the motor network are unconstrained. Across several subjects, we see selective engagement and differential activation based on the task being performed. For example, in Subject 4, we see maximal activity in the dorsal pre-motor area during the kinesthetic imagery screening task, and parietal engagement only when feedback is provided (figure 6). This demonstrates that sEEG allows for the assessment of learning and adaptation on the network level, and that cortical subnetworks were differentially engaged across tasks.

## 4. Discussion

### 4.1. Contextualizing the findings

We demonstrate that successful control (⩾80% accuracy) of an external cursor from pericentral depth electrodes is possible in cortex and at the gray–white junctions across all subjects (except subject 24, who had only a white matter control site) during overt movement. While overt movement-based control was possible from motor association areas away from the central sulcus, control using kinesthetic imagery is more feasible with more proximity to the PCG. The control channels in nine of the eleven successful imagery BCI conditions were in the PCG (positions shown in figure 5). The lack of BCI control using kinesthetic imagery from regions away from the central sulcus might be explained by minimal task engagement by the patient in response to increased difficulty, insufficient opportunity to learn (often due to the rapidly progressing clinical schedule), or an inability of the motor circuitry to adapt to the feedback. Instances of poor control driven by attentional lapses or fluctuations in participant engagement are not easily captured by neurophysiology alone. Due to this, they must be identified by the experimenter through behavioral cues, such as body posture or eye movement.

Across the learning process, we see that broadband gamma power is separated into two distinct sub-distributions (figure 4), with the active targets being more easily hit than passive targets early on in learning. Presumably, this may be due to the more concrete nature of kinesthetic imagery performance that allows subjects to anchor to a tangible process. This may be explained by the fact that passive feedback conditions require lowering broadband power (approximating cortical activity) at the control channel, which is much less natural compared to cortical engagement. This explanation is supported by results in subject 5 where the combination of imagined tongue and hand movement led to increased accuracy compared to imagined hand movement paired with rest (figure 5).

Imagery BCI using hand movement alone was successful in ten out of seventeen conditions. In contrast, those conditions involving tongue or foot—even if hand movement controlled one direction (e.g. figure 5, Subject 6)—were successful in only two of nine conditions. This may indicate that the hand is the best control effector for BCI, but a larger sampling of BCIs not involving the hand is needed to test the robustness of these differences.

Almost all (ten out of twelve) control channels in the successful imagery BCI experiments were located inside the PCG. The exceptions were subjects 16 and 23, whose control channels were in the operculum near the central sulcus and cingulate, respectively (table 1, figure 5). Selection of control channels within the PCG does not guarantee success, as six out of thirteen (46%) control channels of the unsuccessful imagery BCI experiments were located in the PCG (table 1). In subject 7, the control channels could not be localized due to aberrant anatomy secondary to a perinatal stroke. Neither of the channels in subject 7 led to successful imagery-based control, but this is a case where we have the opportunity to study the physiology in pathological tissue during BCI.

An additional strength of this dataset is the inclusion of both adult and pediatric participants, which allowed us to observe that the general principles of peri-central control and imagery feasibility were consistent across age groups. Although our study was not powered to systematically compare developmental effects, the ability to obtain reliable control signals in younger patients suggests that sEEG-based BCI approaches may be broadly generalizable across the lifespan. Future work with larger pediatric cohorts will be essential to determine whether maturation-related differences in motor-network organization influence BCI learning or performance.

Although training time available for study was limited due to the clinical context, we found no significant correlation between the number of training trials and peak accuracy achieved by each subject (Pearson’s r: −0.045, p: 0.83, see table 1 for data). This suggests that there may be subject-specific differences in their ability to learn imagery BCI, independent of training volume.

Our initial data in a single subject may support the concept that movement, kinesthetic imagery, and imagery BCI differentially engage the motor network (figure 6). To make this claim, a much more detailed exploration, which is outside the scope of this work, would be necessary. Still, the examination of the unique roles of non-primary nodes of the motor network in online learning is a critical advantage of sEEG-based BCI. Future BCIs might exploit these dynamics *across* distributed circuits rather than rely on single-site control.

### 4.2. Learned general principles for brain computer interfacing

The goal of this work is to generate results that establish scientific principles that advance our understanding of brain-computer interfacing and advance the field toward improved patient care.

This simple 1D feedback study establishes a few important basic principles: 1) cursor control from peri-central depth electrodes is possible across all subjects. 2) Imagery-based control is most feasible near the central sulcus, specifically the PCG. 3) Overt movement, kinesthetic imagery, and feedback each recruit discrete yet overlapping networks.

This study shows that movement (i.e. overt) can be decoded widely. Imagery-associated feedback seems to be driven best within the PCG. This would not necessarily have been predicted from the anecdotal comparison of movement-associated change to imagery-associated change (figures 6(B) vs (C)) in which pre-motor sites were more actively recruited during imagined movement without feedback.

Importantly, although imagery-based BCI is a compelling metric for studying BCI in individuals with intact motor systems (as in our clinical cohort), the eventual target users of prosthetic BCIs will often be individuals with impaired but partially preserved motor networks (e.g. poststroke, early ALS) who will rely on attempted overt movement. In such populations, overt movement—rather than kinesthetic imagery—is likely to be the more relevant and practical control strategy. For this reason, performance during kinesthetic imagery BCI should not be overemphasized relative to overt-movement BCI.

While our approach leverages existing cortical activity patterns involving movement and imagined movement, a different approach to BCI might be to give the participant feedback from a random location in a cortical association area, and let them learn the mapping between location and cursor in an operant conditioning approach (i.e. learn location and power implicitly for control [50]). Although we did not have time to attempt this due to clinical time constraints, we have a unique opportunity in sEEG to attempt to map which brain regions can be operantly conditioned to control a cursor at a mesoscale (thousands of neurons).

### 4.3. sEEG’s role among current BCI approaches

Historically, intracranial BCIs range from ECoG grids, which record cortical activity across large populations of neurons, to microelectrode arrays (MEAs), which employ microelectrodes to record the action of single neurons. ECoG grids are safe and provide reliable signals over years, but offer low-dimensional control to users [51, 52]. MEAs enable sophisticated decoding and high-dimensional control [9, 53, 54], but currently lack the scalability to be deployed across a set of patients with diverse clinical needs [55, 56]. sEEG offers the safety of ECoG [57, 58] in conjunction with recordings from the brain’s depths.

sEEG allows access to distinct nodes of hierarchical functional networks. For example, the network involved in grasping an object would include nodes that encode: reaching speed—premotor cortex [59], hand orientation—parietal reach region [60], task valence—anterior cingulate [61], and execution—primary motor cortex. This ability to tap into individual nodes allows for 1) the generation of an assortment of potential control signals and 2) the chance to study the underlying network dynamics during behavior to improve effective implementation of future BCIs. These capabilities will pave the foundation for more nuanced studies aiming for multidimensional control of virtual and physical effectors, speech decoding, or speech intent production. In addition to multiple recording locations, a synthesis of modalities may offer the best BCI for patients [62]. For instance, broadband power changes in the hand knob of the primary motor cortex could be combined with single-unit recordings (e.g. Neuropixel) in premotor cortex.

In addition to high-performance decoding, future BCIs would benefit from the ability to steer circuit activity toward a desirable state, which enhances decoding. A parallel to this is t he RC+S device, which can simultaneously record and stimulate to deliver closed-looped therapy in the context of conditions such as epilepsy or movement disorders [63]. sEEG offers the ability to trial this closed-loop stimulation while patients undergo inpatient seizure monitoring. Although this application may not immediately relate to BCI, whether or not stimulation can guide circuitry into more conducive states for BCI is a principle that should be tested.

As BCIs transition into more patient-centric clinical research, it is important to prioritize signals that ensure high BCI accuracy chronically within healthy and pathologic tissue. Furthermore, these signals may need to be obtained from lesioned brain areas experiencing neurodegeneration or plasticity, such as in amyotrophic lateral sclerosis or stroke. The combination of signals spanning cortical and subcortical structures has the potential to provide the flexibility necessary to allow BCI control across tissue qualities over extended periods.

### 4.4. A new paradigm for individualized BCI implants

High-fidelity, robust clinical BCIs will require solutions that are specific to the individual. The practice of employing sEEG to create a patient-specific treatment already exists in the context of drug-resistant epilepsy. This entails the temporary implantation (days to weeks) of sEEG leads to guide future treatment [12]. Frequently, the results of this screening indicate a need for permanent stimulation devices directed at nodes within each patient’s seizure network. In addition to epilepsy, sEEG monitoring is used for OCD [64] and depression [65]. Based on our study, we propose that evaluation with sEEG could be used to identify targets for a permanent BCI implant with depth electrodes (which themselves may be at a higher-density scale). This may be particularly useful for patients who are nearly locked-in or have atypical anatomy, as past studies have found a lack of ability to control with permanent devices implanted late in ALS [66]. Additionally, sEEG provides direct, electrical evidence of the best sites for permanent BCI control, which is not possible using non-invasive screening modalities such as high-density scalp EEG, functional MRI.

## 5. Conclusion

The ability to interrogate and modulate distributed networks frames sEEG as a powerful complement to penetrating existing BCI approaches. Specifically, we show that 1) cursor control from peri-central depth electrodes is possible across a large set of pediatric and adult subjects, 2) imagery-based control is most feasible near the PCG, and 3) overt movement, kinesthetic imagery, and feedback each recruit discrete yet overlapping networks. Exploiting this volumetric access may therefore accelerate translation from laboratory demonstrations to scalable clinical neuroprostheses.

## Supplementary Material

Supplementary Material

Supplementary material for this article is available online

Supplementary Figures available at https://doi.org/10.1088/1741-2552/ae2955/data1.

## Figures and Tables

**Table 1.** Subject Information. Subject ID, age/sex, number of recording electrode contacts/number of leads/side, BCI modality, anatomy, accuracy in overt BCI across final 2 runs, accuracy in imagery BCI across final 2 runs, Number of trials performed by the subject prior to their final 2 runs of imagery BCI, regardless of success rate.

ID	Age/Sex	Hand	No. Elecs/Leads/Side	BCI Modality	Anatomy	Accuracy Overt	Accuracy Imagery	Learning Trials

1	10/F	R	198/14/L	—	—	—	—	—
2	16/F	L	217/14/B	T/ RF	PCG	100	93	79
3	17/F	R	168/13/R	LH	PCG	95	97.5	69
4	18/M	L	231/14/R	LH	PCG	100	89.5	32
5	15/F	R	159/10/L	RH	PCG	100	50	19
5	15/F	R	159/10/L	RH/T	PCG	95	75.5	134
6	13/M	R	196/13/R	H/F	PCG/CIN	100	73	24
7	15/M	L	185/12/L	RH	AA	100	47.5	42
7	15/M	L	185/12/L	RH	AA	83.5	50	93
8	36/F	R	199/14/B	T/F	PCG	100	45	34
8	36/F	R	199/14/B	H/F	PCG	96	86	18
9	8/F	R	230/17/B	RH	PCG	90	—	—
10	19/M	R	211/15/B	RH	CS*	100	36	19
11	6/M	R	193/13/R	LH	PCG	100	—	—
12	37/M	R	237/15/B	T	CS/PoCG	100	45	0
13	15/M	R	215/16/B	LH	PCG	100	100	0
14	35/M	R	252/15/B	RH	PCG	100	100	26
15	17/F	R	232/15/B	RH	PCG	90.5	—	—
16	36/M	B	195/15/R	LH	OPC	100	84	143
17	12/F	R	232/16/B	LH	CS*	100	66	9
18	16/M	R	254/16/R	LH	PCG	96	100	6
19	46/F	L	256/13/B	RH	PCG	100	55	0
20	31/F	L	236/15/B	LF	PCG/CIN	94	74.5	41
21	16/M	R	241/16/B	RH	PCG	100	100	0
22	15/M	L	218/15/L	T	SCL/PoCG	100	76	26
23	16/F	R	255/15/B	RH	PCG/CIN	85	84	0
24	15/M	R	255/15/B	LH	WM	50	—	—
25	21/M	B	201/12/L	RH	PCG	91	—	—
26	18/M	R	230/16/L	RH	PCG*	96	90.5	0
26	18/M	R	230/16/L	RH	PCG	61.5	—	—
27	15/F	R	166/13/R	LH	PCG	100	—	—
27	15/F	R	166/13/R	LF	PCG	100	61	41

L/R = left/right, H = hand, T = tongue, F = foot, PCG = precentral gyrus, CIN = cingulate gyrus, AA = aberrant anatomy, CS = central sulcus, PoCG = postcentral gyrus, OPC = operculum, SCL = subcentral sulcus, WM = white matter, * = white/gray matter boundary (supplementary figure 2), - = did not participate, ‘/’ indicates that modalities pushed cursor in opposing directions.

## Data Availability

The data that support the findings of this study will be openly available following an embargo at the following URL/DOI: https://osf.io/kxqvw/. All code will be made available at the author’s GitHub https://github.com/michaeljensen42/Feasibility_stereo_EEG_BCI.
