# Supplementary material for: sEEG-based brain-computer interfacing in a large adult and pediatric cohort
Source: J Neural Eng. Author manuscript; Available in PMC 2026 Apr 29. (PMC13127260; doi:10.1088/1741-2552/ae2955)
Supplement: Supplementary Material [file NIHMS2158990-supplement-Supplementary_Material.docx]

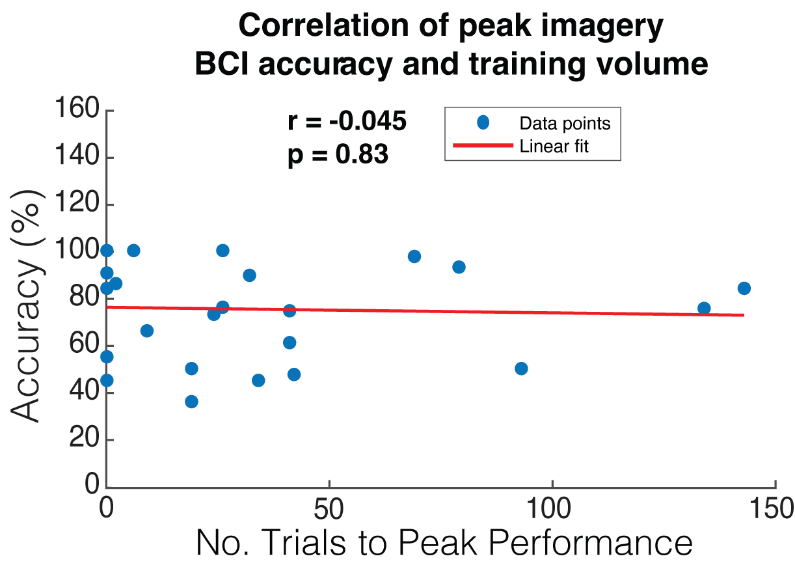
**Supplementary Figure 1. Number of Trials to Peak Performance – Motor Imagery.** Each blue circle represents the peak performance for each imagery BCI experiment across all subjects. The red line is the linear fit with pearson correlation and p-value show above. As seen, the number of trials until peak performance is not a strong predictor of peak performance.


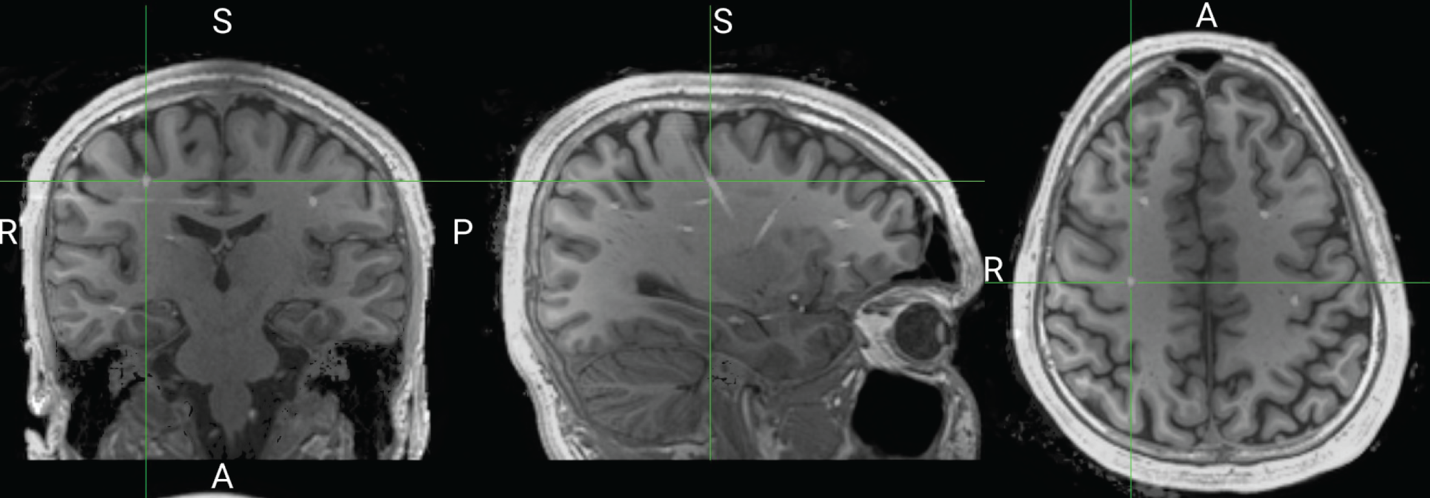


**Supplementary Figure 2. Example of a control channel (Subject 10) in the gray-white matter boundary, Subject 10.** A sEEG electrode at the edge of the cortical gray matter of the central sulcus is shown within coronal (left), sagittal (middle), and axial (right) slices via co-registered post-operative CT and pre-operative MRI files. The green crosshairs highlight this electrode. With this visualization, it is clear to understand how a bipolar channel consisting of the highlighted channel and the next most distal electrode would be labeled as a gray-white channel.


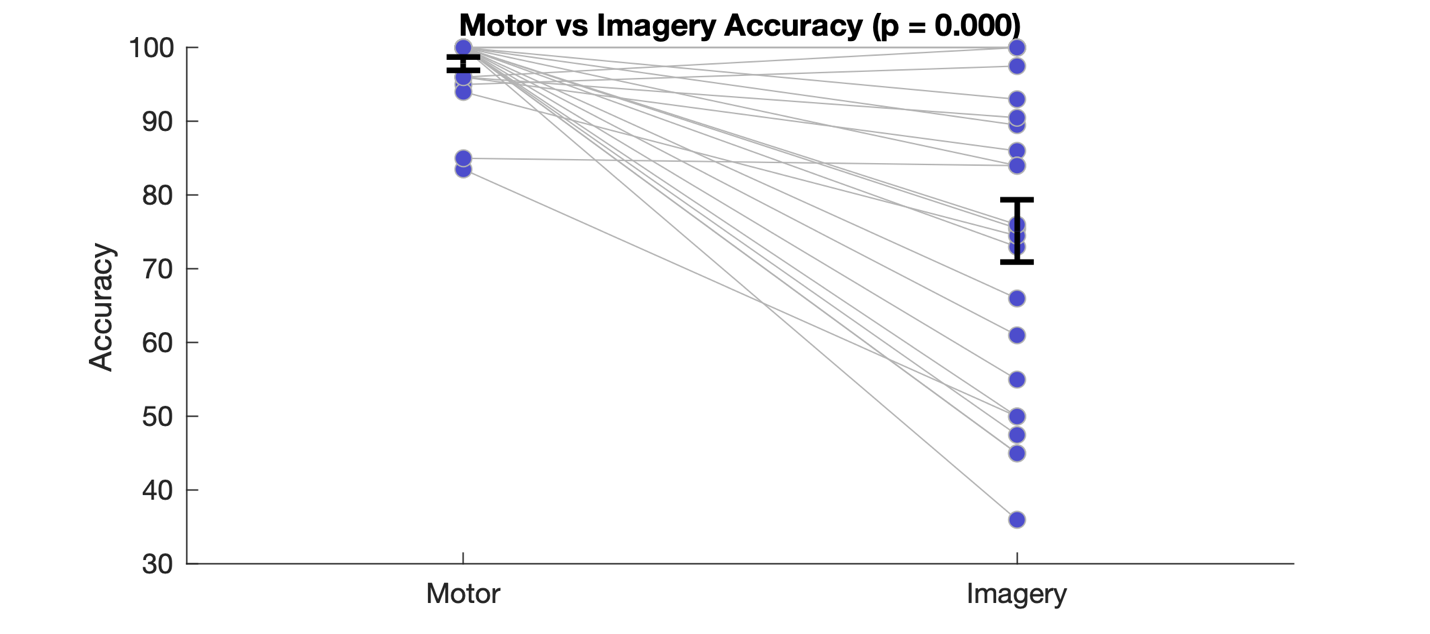


**Supplementary Figure 3.** Comparison of motor and imagery BCI accuracies across subjects. Each line connects the motor and imagery accuracy for an individual subject. Mean accuracy ± SEM is shown for each condition. Imagery performance was significantly lower than motor performance (paired Wilcoxon signed-rank test, p = 0.000).


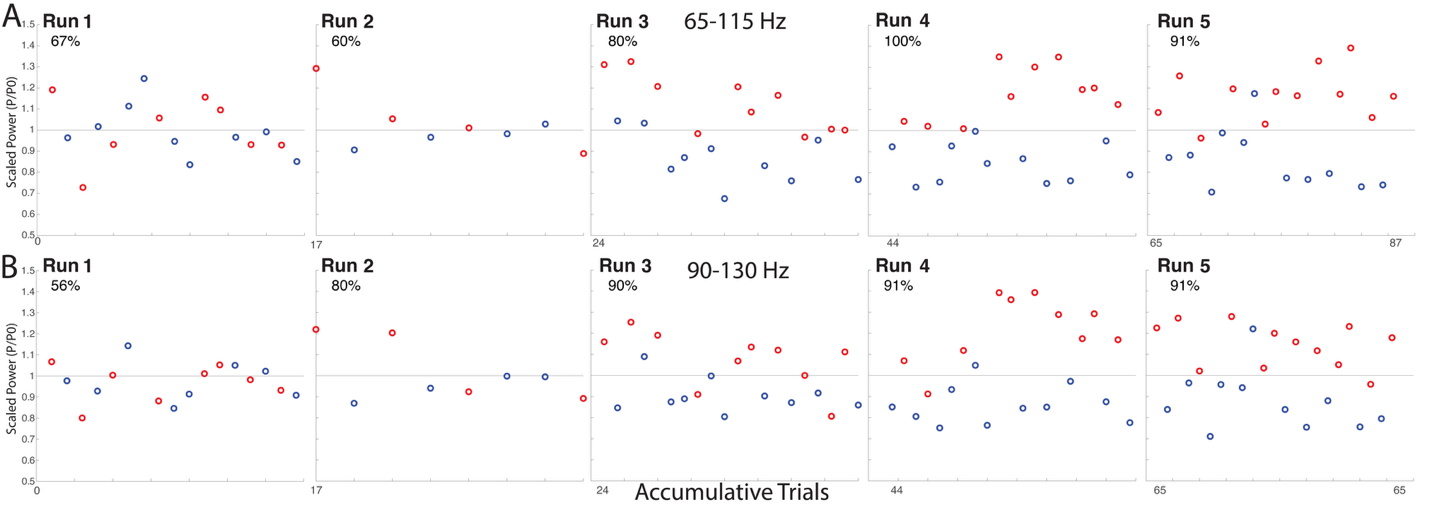


**Supplementary Figure 4.** Comparison of trial-by-trial high-frequency broadband (HFB) power calculated over 65–115 Hz versus 90–130 Hz. **A.** Each point represents 65-115 Hz power within a single trial while the cursor is being controlled (accuracies listed below each run number). **B.** Each point represents 90-130 Hz power within a single trial while the cursor is being controlled. Together, these demonstrate that while the choice of HFB range does impact accuracy, the effect is minimal. It also demonstrates that using bipolar re-referenced data, 90-130 Hz power can be resistant to background line noise at 120 Hz (in U.S).
